# Quantitative and correlative extreme ultraviolet coherent imaging of mouse hippocampal neurons at high resolution

**DOI:** 10.1126/sciadv.aaz3025

**Published:** 2020-05-01

**Authors:** Peter D. Baksh, Michal Ostrčil, Magdalena Miszczak, Charles Pooley, Richard T. Chapman, Adam S. Wyatt, Emma Springate, John E. Chad, Katrin Deinhardt, Jeremy G. Frey, William S. Brocklesby

**Affiliations:** 1 University of Southampton, Zepler Institute, Southampton, SO17 1BJ, UK.; 2 RWTH Aachen University, Experimental Physics of EUV, JARA-FIT, Steinbachstrasse 15, 52074 Aachen, Germany.; 3 Dipartimento di Ingegneria dell’Informazione, University of Padova, Dei, Via Gradenigo, 6B - Padova, Italy.; 4 Department of Chemistry, University of Southampton, Southampton, SO17 1BJ, UK.; 5 Central Laser Facility, STFC Rutherford Appleton Laboratory, Harwell Campus, Didcot, Oxon OX11 0QX, UK.; 6 Clarendon Laboratory, Department of Physics, University of Oxford, Oxford OX1 3PU, UK.; 7 University of Southampton, Biological Sciences, Southampton, SO17 1BJ, UK.

## Abstract

Microscopy with extreme ultraviolet (EUV) light can provide many advantages over optical, hard x-ray or electron-based techniques. However, traditional EUV sources and optics have large disadvantages of scale and cost. Here, we demonstrate the use of a laboratory-scale, coherent EUV source to image biological samples—mouse hippocampal neurons—providing quantitative phase and amplitude transmission information with a lateral resolution of 80 nm and an axial sensitivity of ~1 nm. A comparison with fluorescence imaging of the same samples demonstrated EUV imaging was able to identify, without the need for staining or superresolution techniques, <100-nm-wide and <10-nm-thick structures not observable from the fluorescence images. Unlike hard x-ray microscopy, no damage is observed of the delicate neuron structure. The combination of previously demonstrated tomographic imaging techniques with the latest advances in laser technologies and coherent EUV sources has the potential for high-resolution element-specific imaging within biological structures in 3D.

## Teaser

Imaging using coherent extreme ultraviolet radiation from laser sources provides high-resolution images of biological samples.

## INTRODUCTION

X-ray imaging has been influential in the study of biological systems since its first demonstrations in the late 19th and early 20th centuries, providing the ability to see within thick samples and the potential for very high resolution due to short wavelengths. Advances in source brightness and computational techniques have resulted in substantial improvements in x-ray imaging, with the most radical changes resulting from the recent development of coherent x-ray sources, which can be used to implement lensless imaging techniques. Coherent x-ray radiation is not widely available and mostly limited to large facilities such as synchrotrons or free-electron lasers. However, in the soft x-ray and extreme ultraviolet (EUV) spectral regions, coherent radiation is available via nonlinear optical techniques and, in particular, from high harmonic generation (HHG) using intense femtosecond lasers (*1*). Radiation in this spectral region can provide high spatial resolution imaging with reduced sample damage but retain the ability to image deep within thick structures, unlike electron-based imaging techniques.

Lensless imaging, also known as coherent diffractive imaging, overcomes the problems inherent in making x-ray optics suitable for microscopy. In a lensless imaging experiment, the object is illuminated with coherent radiation, and the scattered radiation is collected onto a detector without the use of imaging optics. The image is created by algorithmic reconstruction of the phase of each pixel of the detected scatter pattern (*2*), allowing direct mathematical transformation between scatter pattern and real space image. The advantages of lensless imaging include the absence of lens aberrations, a lateral resolution limited only by collected numerical aperture rather than detector pixel size or focal spot size, and the ability to reconstruct the intensity and phase of radiation transmitted through the sample rather than intensity alone. However, lensless imaging methods generally have stringent requirements of high stability and spatial coherence of the illuminating beam. HHG sources can, in principle, meet these requirements, allowing coherent imaging in the EUV to be performed in a small-scale laboratory (*3*–*8*).

Theoretically, lensless imaging promises diffraction-limited resolution; however, in practical applications, resolution is often limited by low signal or systematic errors, particularly when using HHG radiation where flux and stability are low compared to synchrotron sources. Ptychography, a form of scanning lensless imaging in which the illumination is moved relative to the sample and multiple scatter patterns are recorded, uses algorithms that are more robust against noise. However, resolution is still limited by both signal-to-noise ratio of the collected scatter patterns and the stability and coherence of the source. As a result, previous demonstrations of ptychography (*4*–*8*) using HHG have obtained high spatial resolution when imaging high contrast or test samples. For HHG ptychography to be used in biological applications, all of the problems caused by low signal level, limited detector dynamic range, source stability, and coherence must be solved. The improved source parameters and reconstruction algorithms used in our measurements allow imaging of biological samples, overcoming many of these problems.

In this work, we demonstrate ptychographic coherent imaging of biological samples with a lateral resolution of 80 nm, which is close to the diffraction limit, and axial sensitivity equivalent to a layer of protein of thickness ~0.8 nm, with a HHG-based source at a wavelength of 29 nm (43 eV). The high lateral resolution is a result of the short wavelength and the accuracy of the phase retrieval algorithm. The high axial sensitivity is due to the very strong interaction of the EUV with materials—a 10-nm layer of protein absorbs 22% of light at 29 nm and produces a 0.15 radian phase shift. Correlated EUV and visible fluorescence imaging of stained samples are performed to demonstrate that EUV transmission can image very thin structures that are not visible in fluorescence-based measurements because of the very small amounts of material present and thus can help in interpretation of visible fluorescence images of the biological structures. No damage is observed in the samples on repeated irradiation, in contrast to the effect of hard x-ray irradiation.

## RESULTS

Mouse hippocampal neuron samples cultured on silicon nitride membranes were prepared as described in Materials and Methods and grown either for 7 or 14 days in vitro (7DIV or 14DIV). The resulting samples were imaged using an optical microscope, both before and after the fixing process. Ptychographic imaging of small areas of the samples was then performed using coherent 29-nm (43 eV) radiation produced using HHG, as detailed in the “Experimental Imaging Techniques” section.

**Fig. 1:**
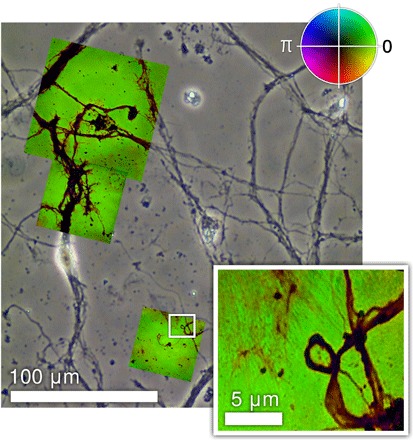
Overview image of 7DIV neuron sample, using visible and EUV radiation. Monochrome image is a visible light transmission image of the neuron sample before fixing. Colored images are from high-resolution 29-nm EUV transmission taken using ptychography: Image intensity corresponds to transmitted amplitude, and image color shows the transmitted phase with values indicated by the inset color wheel. The image inset shows detail of one of the EUV images containing fine structure present in the ptychographic reconstruction but not present in visible light image, demonstrating the enhanced resolution achieved with EUV light.

An overview image of a 7DIV neuron sample is shown in Fig. 1. The monochrome background shows a white light phase contrast optical microscope image of the whole sample. The colored insets are three EUV transmission images, taken using ptychography. The white light image was taken before the sample was fixed for use in the vacuum chamber. Each of the EUV images shows a specifically chosen small region of the sample at high resolution. In these images, the intensity represents the transmitted amplitude, and the false color represents the transmitted phase of the EUV radiation passing through the sample as indicated by the color wheel in the figure. The optical images illustrate the context of the smaller EUV-imaged regions with respect to the cell bodies and allow more accurate identification of structures within the EUV images.

**Fig. 2:**
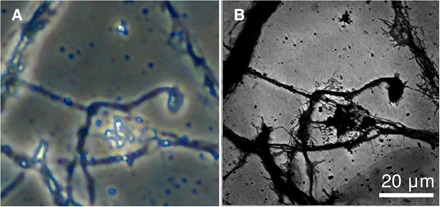
Comparison of optical and EUV images. Figure shows an 85-μm area of the 7DIV neuron sample: (**A**) White light phase contrast microscope image of sample after fixing; (**B**) 29 nm (43 eV) EUV intensity transmission using ptychography of sample after fixing. The extra detail and resolution provided by EUV imaging are immediately apparent from the visibility of fine structures around the thicker bundles.

Figure 2 shows a direct comparison of optical and EUV imaging of the same area of a 7DIV sample. Figure 2A is a phase-contrast image of the sample before fixing, taken using a phase-contrast visible light microscope. Figure 2B is the EUV transmission function of the same sample obtained using ptychographic imaging with a 29-nm radiation. The EUV image is reconstructed from 1729 scatter patterns. The much higher lateral resolution of the EUV image over the optical image is evidenced by resolving the fine “flared” structures emanating from the thicker dendrites in the former image that are not present in the latter. The increase in lateral resolution arises from the use of the 27th harmonic of the laser (780 nm/27 = 29 nm) for imaging, reducing the diffraction limit by an equivalent factor. The EUV image pixel size in this case is 137 nm, determined by the numerical aperture of the data collection geometry, which can be varied between experiments. Increasing the experimental numerical aperture reduces pixel size, as demonstrated in the next section, where a change in detector geometry reduces the pixel size to 58 nm and increases the resolution as a result.

### Lateral resolution

**Fig. 3:**
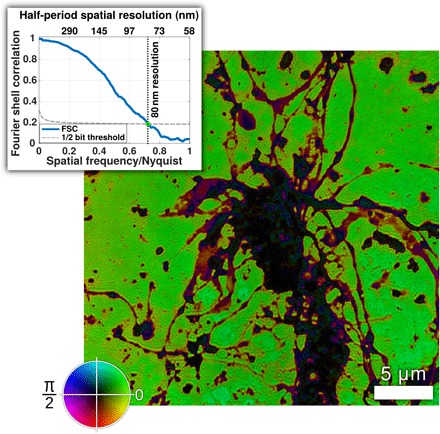
Lateral resolution determination by FRC. Amplitude and phase representation of a region of a 7DIV neuron sample. As in Fig. 1, image intensity represents object amplitude transmission, and image color represents phase, with the same color scale used in Fig. 1. Inset shows the FRC function versus spatial frequency between reconstructions of two independent datasets from the same sample area. The effective resolution as demonstrated by the FRC is 80 nm.

The lateral resolution of the reconstructed images of real-world biological objects is more complex to measure than in equivalent experiments on man-made test samples (*5*, *7*), because man-made samples typically have features with well-defined sizes and shapes, whereas biological objects do not. As the best indicator of resolution, we use the Fourier ring correlation (FRC) function, the two-dimensional (2D) equivalent of the Fourier shell correlation function (*9*, *10*) used in x-ray and electron microscopy to estimate resolution. The FRC measures the correlation between two independent images of the same sample area as a function of spatial frequency. To get two independent images, the scatter data were split into two separate datasets, by assigning scatter patterns from different sample translation coordinates to either one or the other data set. Each data set is then reconstructed separately. The two resulting images can be used to create the FRC. Figure 3 shows the complex EUV transmission of a second mouse neuron sample also grown for 7DIV. The image was reconstructed from 251 scatter patterns, taken using an experimental data collection geometry, which produced a pixel size of 58 nm rather than the 137-nm pixel size seen in Fig. 1. The image color indicates the phase of the complex transmission, using the scale shown by the color wheel. The inset in the figure shows the FRC as a function of spatial frequency. The solid line is the FRC, and the dotted line represents the half-bit information level suggested by van Heel and Schatz (*11*) as a general-purpose indicator of interpretable resolution. The FRC is expressed in terms of the Nyquist frequency of the image. Here, the FRC reaches the half-bit level at 0.7 of the Nyquist frequency, indicating a half-period resolution of 80 nm, 38% larger than the diffraction-limited half-period resolution (pixel size) of 58 nm.

### Quantitative phase measurement and axial resolution

Unlike traditional x-ray imaging, ptychographic imaging measures both the attenuation and the phase shift caused by the object, as well as the full electric field profile of the probe light illuminating the object. The refractive index *n* of the object material in the EUV can be represented by *n* = 1 − δ − iβ, where δ determines the phase shift through the medium, and β determines the intensity attenuation coefficient, μ. The measured attenuation, *I*/*I*_0_, and relative phase shift, ϕ, across the sample are simply related to δ, β and the sample thickness, *z*, via

$$\frac{I}{I_{0}}=\text{exp}(-\mu z)=\text{exp}[-2(\frac{2\pi }{\lambda }\beta )z]$$ (1)

and

$$\phi =-\frac{2\pi }{\lambda }\delta \;z$$ (2)

where *I* is the transmitted intensity, *I*_0_ is the incident intensity, and λ is the wavelength of the illuminating radiation. Hence, δ × *z* and β × *z* can be calculated directly from the ptychography data. The attenuation and relative phase shift of the thin film of silicon nitride supporting the sample can be calculated from the transmission in regions of the sample for which there is no biological material, allowing the contribution from the neurons to be isolated.

**Fig. 4:**
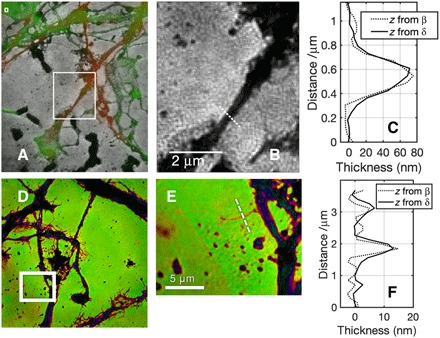
Cross-sectional measurements of stained and unstained neurites. (**A**) Monochrome image is the EUV intensity transmission of a 7DIV neuron sample. Overlaid are wide-field immunofluorescence images of the same sample showing actin (green) and tubulin (red). (**B**) EUV intensity transmission image or the region shown by the white box in (A), showing a neurite known to be tubulin-enriched. (**C**) Cross sections of the feature along the white dotted line derived from the amplitude and phase of the EUV image, using the complex refractive index of tubulin. The thicknesses agree to within 8%. (**D**) Amplitude and phase representation of another, unstained 7DIV sample. (**E**) Magnified region within the white box of (D), showing a small (300 nm width) protrusion from another neurite. (**F**) Cross section along white dotted line in (E), showing calculated feature heights using real and imaginary parts of the generic protein refractive index. The heights agree to 12%.

To test the quantitative nature of the transmission function, we can extract values of δ × *z* and β × *z* and compare these two values based on expected sample composition. Figure 4A shows the intensity transmission function of a ~5 μm × 5 μm region of a sample of 7DIV neurons that have been stained for immunofluorescence imaging. The white box shows the exact position within the larger area where the magnified image shown in (B) is located. In (A), the monochrome image represents EUV intensity transmission, and the red and green overlays are correlated tubulin and actin optical fluorescence images. The magnified image in (B) shows a neurite, which can be identified as being enriched in tubulin from the correlated fluorescence image in (A). Figure 4C shows a cross section of the neurite thickness, taken along the white dotted line. In this figure, the values plotted are the sample thickness *z*, derived separately from either the real part of the measured transmission function δ × *z* (solid line) or from the imaginary part β × *z* (dotted line). The values of δ and β used here are those for mouse tubulin, based on the molecular formula from the ProtParam tool (*12*) and density given by Howells *et al*. (*13*). The values of thickness derived from real and imaginary parts of the EUV refractive index agree to within 8%, based on the identification of the feature as tubulin from correlative fluorescence imaging.

Figure 4D shows a ~86 μm × 86 μm region of a different 7DIV sample, with color scale representing amplitude and phase of the transmission as in Fig. 1. Figure 4E shows a magnified image of the region enclosed by the white box in (D), showing a 300-nm-wide protrusion from one of the neurites. No composition information is available directly because this sample was not prepared for immunofluorescence. Figure 4F shows a cross section of the sample thickness along the white dotted line in (E). Thicknesses are calculated by separately using the real and imaginary parts of the EUV, as for (C), based on the composition of the protrusion being protein. The thicknesses agree to 12%, supporting the identification of the feature as being composed of protein. Over large sample areas, the root mean square (RMS) noise in intensity is 3.3%, and the RMS phase noise is 0.02 rad, indicating that the intensity noise floor is equivalent to 1.3-nm thickness of the protein layer, and the phase noise floor is equivalent to 0.8 nm in the same material.

### Correlative EUV and fluorescence imaging

**Fig. 5:**
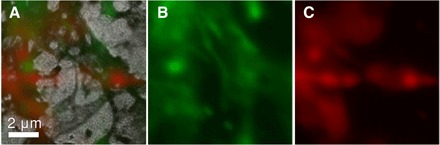
Correlative EUV/fluorescence imaging. Image (**A**) shows the EUV transmission intensity, with superposed actin (green) and tubulin (red) images from immunofluorescence wide-field fluorescence microscopy. (**B**) and (**C**) show the individual fluorescence images. The increase in resolution and the extra detail observable in the EUV transmission image are immediately clear.

Several neuron samples that had been stained for immunofluorescence imaging were prepared for correlative imaging using both EUV ptychography and conventional fluorescence microscopy (as described in Materials and Methods). Figure 5 shows the same sample area imaged with EUV ptychography and with widefield fluorescence microscopy. Figure 5A shows a grayscale image of the EUV intensity transmission of the sample, with a scale bar of 2 μm. Overlaid onto this image are the corresponding fluorescence images of the tubulin stain in red, and the actin stain in green, shown separately in (B) and (C), respectively.

The correlative imaging demonstrates several important points regarding the effectiveness of EUV ptychography in bio-imaging. First, the resolution is clearly significantly better, as would be expected given the shorter wavelength. Second, the EUV is much more sensitive to thin structures in the neurons, where the total amount of fluorescent material is so small that the features are not observable. The ability of the EUV imaging to aid interpretation of the fluorescence images is seen clearly in Fig. 5C, where the actin image could easily be interpreted as having a continuous structure running horizontally across the image just below its center. The EUV image shows very clearly that the underlying structure is not continuous. Correlative imaging of this kind allows the structural elements visible using EUV ptychography to be directly correlated to biological function.

### Radiation damage

Many other high-resolution imaging techniques for biological samples, such as cryo–electron (*14*) or hard x-ray microscopy (*13*) are greatly limited because of the damaging effects of the radiation. Over the course of imaging of neuron samples using 29-nm EUV radiation, no damage effects on the fixed, room temperature samples used in these experiments have been observed. During collection of data for a single reconstructed image, the sample receives a dose of ~10^7^ Gy. At our present signal-to-noise ratio, there is no observable change in the sample image after repeatedly collecting images from overlapping regions on a single sample. This is in contrast to the effect of 7-keV synchrotron radiation on similar samples, where significant damage has been observed with lower dosage (*3*). This indicates that the trade-off between radiation damage and signal-to-noise ratio, which limits resolution with higher energy radiation, is much more favorable in the EUV.

## DISCUSSION

High-resolution and high-quality reconstructed images of unstained biological samples, with features only 10 nm thick, can be produced using a laboratory-scale EUV source via coherent imaging. The images are quantitative in both amplitude and phase; the lateral resolution is close to the diffraction limit, and the axial sensitivity is subnanometer. Correlative imaging has demonstrated how the EUV transmission images can be used to interpret structure seen in fluorescence microscopy in stained samples, and conversely, the information available from immunofluorescence staining, such as direct confirmation of composition, can be used to aid in interpretation of the EUV images. In contrast to hard x-ray microscopy of similar samples, no damage due to the EUV radiation is observed on the exposure timescales used for imaging.

EUV imaging of biological structures provides information not available from fluorescence microscopy alone. Even with the use of superresolution techniques based on fluorescence, such as photoactivated location microscopy (PALM) (*15*) or stimulated emission depletion (STED) (*16*), which can have lateral resolution smaller than the diffraction limit, fluorescence imaging of very thin structures will remain very difficult, because of the extremely small quantities of fluorescent material present. As sources develop, the ability to tune the EUV wavelength to match the attenuation length of the material of interest will provide the option to optimize sensitivity to structures of varying thicknesses, and imaging at wavelengths on either side of an x-ray absorption edge will provide intrinsic contrast. As the attenuation length becomes longer, EUV and soft x-ray ptychography will be able to produce 3D images of structures within the material. Microscopy using laboratory-scale coherent EUV sources can become a valuable complementary technique to other microscopy methods in biology because it can provide high-contrast, label-free imaging at high resolution at a size scale relevant to subcellular molecular organization and biological function.

## MATERIALS AND METHODS

### Neuron sample preparation

Hippocampal neurons were isolated from embryonic day 17 mice and plated onto 50-nm-thick silicon nitride (SiN) membranes following standard protocols. The SiN membranes are prepared by sterilization, followed by coating with poly-l-lysine. The hippocampal neurons were grown in neuronal media (Neurobasal, 2% B27, 0.5 mM glutamine) for 7 or 14 days before fixation in 4% paraformaldehyde, followed by 100% methanol. Some of the samples of 7DIV neurons were stained using immunofluorescence staining with an anti-tubulin antibody and phalloidin to highlight actin filaments, to compare the actin and tubulin structures revealed by fluorescence microscopy with the structures seen in EUV images.

### Experimental imaging techniques

Visible microscope images were taken on an EVOS XL Core microscope, using a ×10 LPlan phase objective. Confocal fluorescence microscope images were obtained with an AP DeltaVision microscope. EUV experiments were performed in the HHG labs at the University of Southampton, United Kingdom and also at the Artemis laser facility, Rutherford Appleton Laboratories, Harwell, United Kingdom. A schematic of the experimental apparatus is shown in the Supplementary Materials. Coherent EUV illumination was produced by a nonperturbative nonlinear interaction of a loosely focused femtosecond laser pulse with argon gas to generate high harmonics of the laser frequency. Pulses with an energy of ~2 mJ, a duration of 40 fs, a repetition rate of 1 kHz, and a central wavelength of 780 μm were focused to a ~100-μm spot in a cell of argon gas at pressures of 50 to 150 mbar. The resulting EUV beam has high spatial coherence, low (few mrad) divergence, and maximum intensity at 29 nm (43 eV) wavelength. The EUV beam is spectrally filtered by transmission through Al foil and focused onto a 10-μm-diameter aperture by a single spherical multilayer mirror, producing the illuminating probe beam. The sample is placed between 50 and 100 μm after the 10-μm-diameter aperture. The flux on the sample is typically 2 × 10^8^ photons/s.

Scattered radiation is collected in the far-field regime using an EUV-sensitive in-vacuum charge-coupled device (CCD) camera placed between 25 and 40 mm after the sample. More details about the ptychography setup can be found in (*6*), and a schematic diagram of the experimental apparatus is given in the Supplementary Materials. The probe illumination was moved by 3 μm between exposures, 70% of the probe diameter. Because of the relatively weak scattering of the sample and low bit depth of the CCD camera, three exposures were recorded at different exposure times between 0.1 s (100 EUV pulses) and 15 s (1.5 × 10^4^ EUV pulses) at each sample position. The resultant images were combined to extend the dynamic range of each measured scatter pattern up to ~17 bits and a maximum measured intensity of ~2 × 10^7^ counts. The limited dynamic range is one of the main challenges for imaging of weakly scattering samples.

### Reconstruction methods

Standard ptychography methods (*2*) reconstruct a complex valued object, *O*(**r**), and a single illumination probe, *P*(**r**), using the collected diffraction patterns, *I_j_*, and the additional information provided by the overlap of the adjacent scanning regions. In the far field and projection approximation, the measured intensity, *I_j_*, produced by a fully coherent beam can be expressed as

$$I_{j}(k)\sim {\left|ℱ[P(r)\cdot O(r-r_{j})]\right|}^{2}$$ (3)

where F is the Fourier transform operator. However, this approximation does not correctly describe an experiment when the illumination changes during the scan, because every scanning position has a slightly different illumination probe. In our experiment, we have used the orthogonal probe relaxation (OPR) method (*17*) that allows us to have a different illumination probe for each scanning position and is particularly appropriate for a source based on HHG, where stability can be low. The variable probe can be expressed as a projection into a low dimensional space

$$I_{j}(k)∼{\left|F[(\sum_{i}U_{i}(r)S_{i}V_{\text{ij}}^{*})O(r-r_{j})]\right|}^{2}$$ (4)

where the matrices U, S, V are given by the truncated singular value decomposition of the current illumination probe *P* estimation, U*_i_* denotes the *i*-th eigenprobe, S is a vector of the eigenvalues, and matrix V is the normalized complex evolution of the eigenprobes. The OPR method provides the additional freedom needed to describe the moving illumination and therefore prevents artifacts in the object reconstruction. For the present reconstructions, two additional constraints have been used to prevent the additional freedom allowed by OPR from reducing probe reconstruction quality. These were the following:

1) Relaxed positivity constraint for the object:

*O* = *O*(1 − α) + α|*O*|, where the relaxation parameter α ≈ 10^−3^.

2) Relaxed support constraint for the eigenprobes:

*U_i_* = P*_x_*{[(1 − α) + α*A*] · P_−*x*_(*U_i_*)}, where α ≈ 10^−2^, *A* is a loose guess of the illumination aperture support and P*_x_* is a propagator into the aperture distance *x*.

The probe support is estimated self-consistently during the ptychography convergence using the idea of the shrink-wrap method (*18*). The relaxed positivity constraint can be used despite the fact that our reconstructions have both phase and attenuation features, because it is only weakly constraining, and it uses the fact that ptychography artifacts usually lead to more phase variation than an artifact-free reconstruction. Coherence properties of the illumination were self-consistently recovered as described in (*4*, *5*).

## Supplementary Material

aaz3025_Movie_S2.mp4

aaz3025_Movie_S1.mp4

aaz3025_SM.pdf

## SUPPLEMENTARY MATERIALS

Supplementary material for this article is available at http://advances.sciencemag.org/cgi/content/full/6/18/eaaz3025/DC1
