# Supplementary material for: Quantitative and correlative extreme ultraviolet coherent imaging of mouse hippocampal neurons at high resolution
Source: Sci Adv. 2020 May 1;6(18):eaaz3025. doi: 10.1126/sciadv.aaz3025 (PMC7195139; doi:10.1126/sciadv.aaz3025)
Supplement: aaz3025_SM.pdf [file aaz3025_SM.pdf]

Science Advances
AAAS

advances.sciencemag.org/cgi/content/full/6/18/eaaz3025/DC1

Supplementary Materials for

Quantitative and correlative extreme ultraviolet coherent imaging of mouse hippocampal neurons at high resolution

Peter D. Baksh, Michal Ostrčil*, Magdalena Miszczak, Charles Pooley, Richard T. Chapman, Adam S. Wyatt, Emma Springate, John E. Chad, Katrin Deinhardt, Jeremy G. Frey, William S. Brocklesby*

*Corresponding author. Email: wsb@orc.soton.ac.uk

Published 1 May 2020, *Sci. Adv.* **6**, eaaz3025 (2020)
DOI: 10.1126/sciadv.aaz3025

The PDF file includes:

Figs. S1 to S3
Legends for movies S1 and S2

Other Supplementary Material for this manuscript includes the following:

(available at advances.sciencemag.org/cgi/content/full/6/18/eaaz3025/DC1)

Movies S1 and S2

H2: Supplementary Materials

Figure S1 - **Schematic of experimental apparatus**

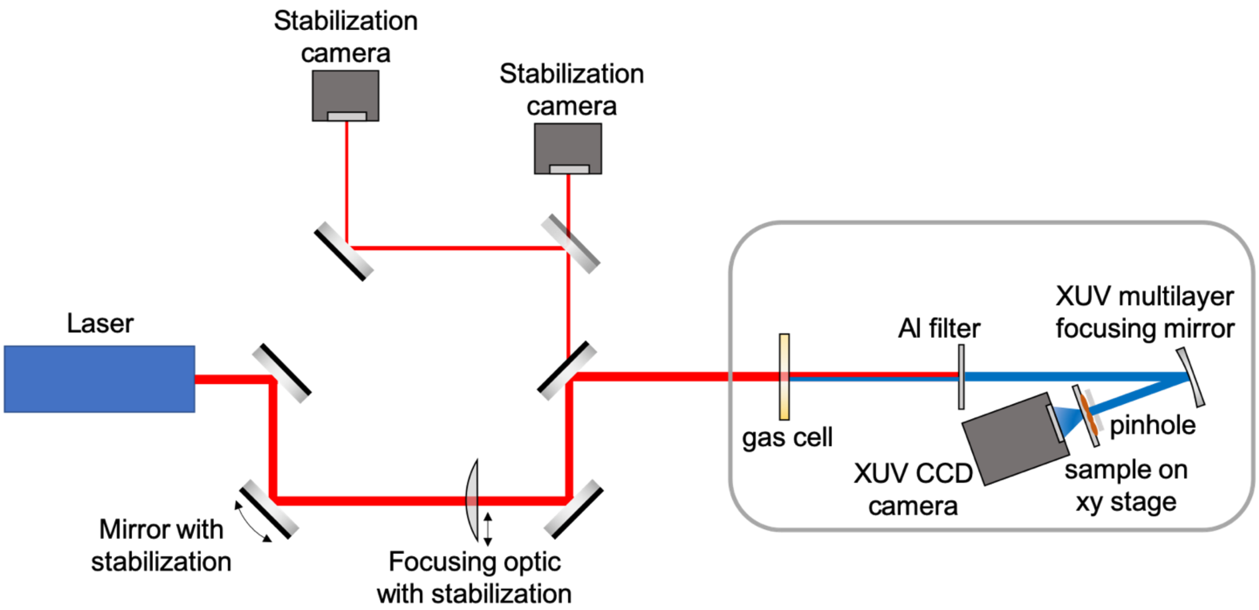

Figure S2**: Raw diffraction image**

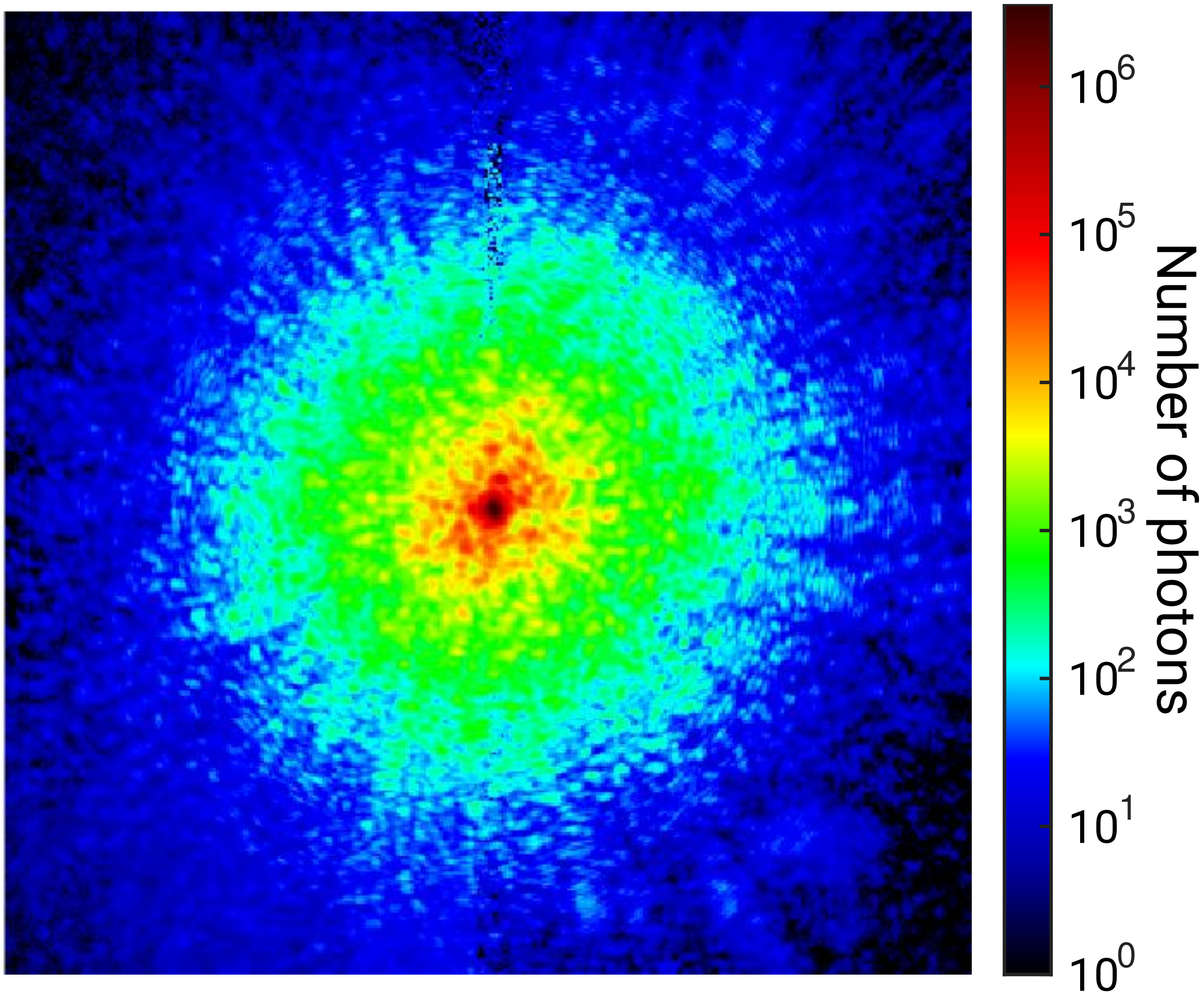

Figure S2 - **Raw diffraction pattern**. This shows an example of the raw diffraction data from which the ptychographic reconstructions are obtained.

Figure S3 - **Probe reconstruction in aperture plane**

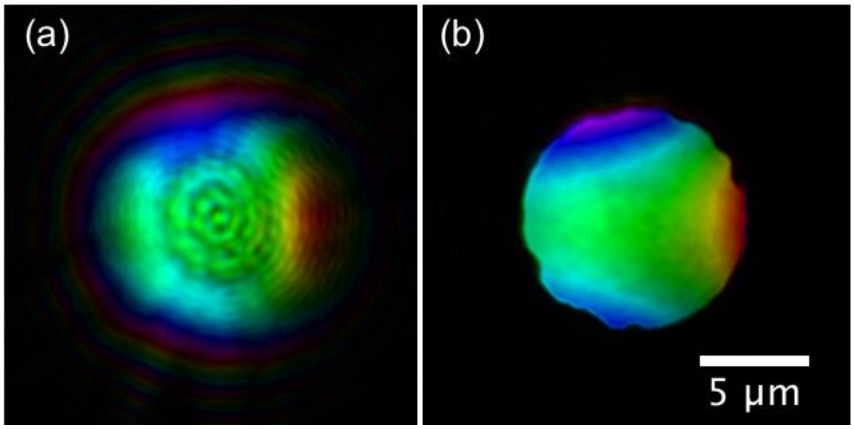

Figure S3 - (a) Complex electric field distribution of the illumination probe at the plane of the sample, reconstructed from data in figure 3 of the paper. (b) Electric field distribution at plane of aperture, numerically back-propagated from data in (a). Saddle-shaped phase of the focused EUV, consistent with the off-axis focusing geometry of the experiment, and the sharp edges of the aperture are clearly visible.

Movies S1 & S2: Probe variation as a function of time

The scattering data are taken sequentially, with each diffraction pattern requiring a time of around 30 s including the time taken to read out the CCD. At each point, the OPR technique calculates the actual probe, which varies slightly from point to point. Thus the temporal variation of the probe during the experiment can be observed by using the calculated probes at each point as frames in an animation. CCD exposure times were ~10 s, with a ~20 s overhead for CCD readout. Thus, some probe changes are seen as jumps rather than a smooth variation.

The animations in the supplementary files show the variation of the probe electric field with time for three different datasets, which produced the images shown in figure 3. As in the figures in the main text, the electric fields shown in the animations are complex, and so we use color to represent the E-field phase, and intensity to represent the E-field amplitude, using the same color wheel as in figure 1 of the paper.

For each dataset, the field variation as measured at the sample is obtained from the ptychography algorithm. By numerically back-propagating the field at the sample, the field variation at the aperture can be calculated, which is easier to interpret. The number of frames in the animation is the same as the number of individual diffraction patterns used to create the final ptychographic reconstruction.

The animations show that there is considerable beam motion during the taking of each dataset, but the OPR technique allows this motion to be decoupled from the image.

Filenames:

Movie S1 filename: probe_anim_sample_dataset_1.m4v

Movie S2 filename: probe_anim_pinhole_dataset_1.m4v

(data in figure 3)
